# Malnutrition Evaluated by CONUT Score and Its Association With Acute Kidney Injury in Acute Myocardial Infarction Patients: A Retrospective Study

**DOI:** 10.1155/crp/1118619

**Published:** 2025-07-10

**Authors:** Masafumi Fukuda, Nobuhisa Hirayu, Masakazu Nabeta, Takehiro Homma, Kei Fukami, Norio Yamashita, Osamu Takasu

**Affiliations:** ^1^Division of Nephrology, Department of Medicine, Kurume University School of Medicine, Kurume, Japan; ^2^Department of Emergency and Critical Care Medicine, Kurume University School of Medicine, Kurume, Japan; ^3^Division of Cardiovascular Medicine, Department of Internal Medicine, Kurume University School of Medicine, Kurume, Japan

**Keywords:** acute kidney injury, acute myocardial infarction, controlling nutritional status, malnutrition, mortality

## Abstract

**Background:** The relationship between nutritional status at the start of treatment for acute myocardial infarction (AMI) and the onset of acute kidney injury (AKI) remains unclear. This study aimed to clarify the association between nutritional status, as assessed by the controlling nutritional status (CONUT) score before catheter treatment, and the development of AKI in patients with AMI.

**Methods:** This retrospective study included AMI patients treated with percutaneous coronary intervention (PCI) immediately after admission at our institution between 2014 and 2018. Patients undergoing chronic hemodialysis were excluded. Nutritional status was evaluated using the CONUT score derived from blood tests at admission, with scores below 5 indicating good nutrition and scores of 5 or above indicating malnutrition. The two groups were compared retrospectively.

**Results:** A total of 211 AMI patients were included, with a median age of 68 years (59–79), and 156 (74%) were male. The median door-to-balloon time was 74 min (59–94). There were 180 patients in the good nutrition group and 31 in the malnutrition group. The malnutrition group exhibited significantly higher mortality (1.1% vs. 12.9%, *p* < 0.001) and a higher incidence of AKI (19% vs. 52%, *p* < 0.001). Multivariable logistic regression analysis identified lactic acid (odds ratio [OR] = 1.570 and 95% confidence interval [CI] 1.310–1.882), baseline creatinine (OR = 7.403 and 95% CI 1.852–29.59), and malnutrition (OR = 3.715 and 95% CI 1.278–10.80) as independent risk factors for AKI.

**Conclusions:** Malnutrition, assessed by the CONUT score before treatment, may be associated with an increased risk of AKI in AMI patients.

## 1. Introduction

Evaluating nutritional status is crucial for understanding the condition of patients. The controlling nutritional status (CONUT) score has recently gained attention as a tool for assessing the nutritional status. The CONUT score has been associated with prognosis in various conditions, including endogenous diseases such as cancer [1], kidney failure [2], heart failure [3], and stroke [4], as well as traumatic conditions like traumatic brain injury [5].

The onset of acute kidney injury (AKI) worsens prognosis. Over 30% of patients admitted to the intensive care unit (ICU) develop AKI, which is associated with poor outcomes in critically ill patients [6]. Acute myocardial infarction (AMI) has a very high mortality rate and remains a serious condition requiring ICU admission, even with advanced medical care [7]. In AMI cases, the development of AKI further increases short-term mortality [8].

There are few reports on the relationship between malnutrition as assessed by the CONUT score and the prognosis of AMI patients [9], and no definitive conclusions have been reached. In addition, the relationship between the nutritional status at the onset of AKI and the development of AKI during treatment is not well understood. This study aimed to clarify the association between the nutritional status before percutaneous coronary intervention (PCI) in AMI patients, their prognosis, and the incidence of AKI.

## 2. Methods

### 2.1. Subjects and Setting

We retrospectively analyzed demographic, clinical, and laboratory data of all AMI patients admitted to the cardiac care unit (CCU) of the Advanced Emergency and Critical Care Center at Kurume University Hospital, Japan, who underwent PCI immediately after admission between January 2014 and December 2018. Data were extracted from medical records and discharge summaries. Patients undergoing regular renal replacement therapy for end-stage renal failure were excluded. The CONUT score was calculated from the serum albumin, total cholesterol concentration, and total lymphocyte count at admission. Patients with a score of less than 5 were classified as the nutritionally sufficient group, while those with a score of 5 or more were classified as the malnutrition group. Statistical analyses were then performed. The protocol for this research project was approved by a suitably constituted Ethics Committee of the institution and conforms to the provisions of the Declaration of Helsinki. The Bioethics Committee for Human Subjects at Kurume University Hospital, Approval no. 22,207, approved the study. The need for written informed consent was waived due to the retrospective nature of the study. The opt-out method to obtain patient consent was utilized at our institution.

### 2.2. Demographic and Clinical Data

Sex, age, body mass index, smoking history, Kidney Disease Improving Global Outcomes (KDIGO) classification [10] of AKI stage, CCU admission days, CCU exit outcome, medication history, comorbidities, and echocardiography were extracted from medical records.

### 2.3. Laboratory Tests

The following blood test results at the start of ICU treatment were obtained from electronic medical records: hemoglobin, hematocrit, white blood cell count, platelet count, lactic acid, glycated hemoglobin, aspartate aminotransferase (AST), lactate dehydrogenase (LDH), total bilirubin, total protein, albumin, blood urea nitrogen (BUN), creatinine, total cholesterol, high-density lipoprotein (HDL) cholesterol, low-density lipoprotein (LDL) cholesterol, triglyceride, hemoglobin A1c, creatine kinase (CK), CK MB, and troponin.

### 2.4. Definition of AKI

The KDIGO criteria [10] were used to categorize patients based on serum creatinine. Baseline creatinine was defined as the serum creatinine at the start of treatment. AKI was defined as an increase in serum creatinine of 0.3 mg/dL or more within 48 h or an increase to 1.5 times or more of the baseline creatinine within 7 days.

### 2.5. Defining Malnutrition

Nutritional status was evaluated using the CONUT score. A CONUT score between 5 and 12 indicates moderate to severe malnutrition and is linked to unfavorable clinical outcomes in hospitalized heart failure patients [11]. According to this classification, patients were divided into two groups: those with a CONUT score below 5 were placed in the nutritionally sufficient group, while those with a score of 5 or higher were classified as the malnourished group.

### 2.6. Statistical Analysis

The design and statistical approach of this study were informed by our previous investigation of AKI in patients with subarachnoid hemorrhage, published elsewhere [12]. Data are expressed as the median and interquartile range (IQR). The Chi-square test was used to compare proportions, while quantitative variables were compared using the Mann–Whitney *U*-test or Student's *t*-test. Variables with *p* < 0.05 from the univariate analysis were entered into a multivariate logistic regression analysis to assess the associations of clinical and laboratory markers with the clinical outcome of AKI. Odds ratios (OR) and 95% confidence intervals (CIs) were calculated. A *p* value < 0.05 was considered statistically significant. Data were missing solely for “onset to balloon time” (*n* = 6); all other variables were fully accounted for. These six cases were excluded only from analyses involving this variable. All statistical analyses were performed using JMP Pro Version 15.1 for Microsoft Windows and SPSS Statistics Pack 27.0 for Microsoft Windows.

## 3. Results

**Figure 1:**
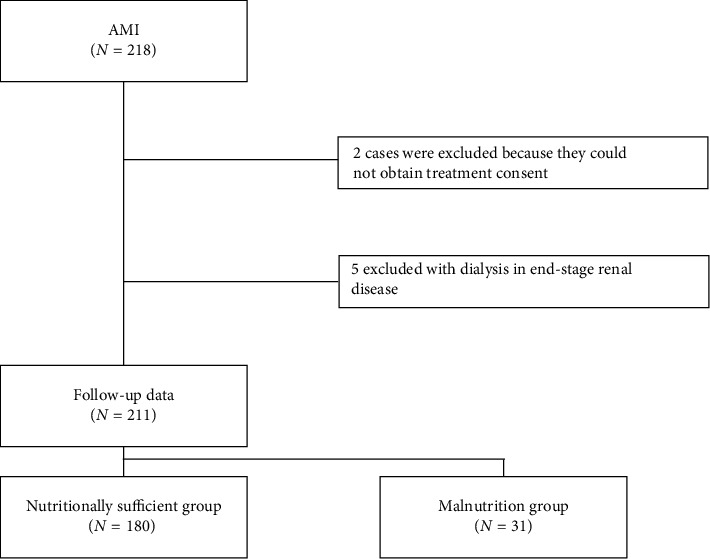
Patient selection scheme. This figure illustrates the classification of 211 patients with follow-up data. Of these, 180 were assigned to the nutritionally sufficient group, and 31 were assigned to the malnutrition group.

A total of 211 patients were included in the study after excluding 5 patients on chronic dialysis and 2 patients who did not receive aggressive intensive care post-PCI due to lack of consent. There were 180 patients in the nutritionally sufficient group and 31 patients in the malnutrition group (Figure 1). Baseline characteristics are shown in Table 1. The median age was 68 years (59–79), and 156 patients (74%) were male. The median door-to-balloon time was 74 min (59–94). The malnutrition group had significantly higher age, AKI incidence, and mortality rates. In addition, the malnutrition group had significantly higher levels of creatinine and BUN and a significantly higher number of patients using calcium channel blockers, beta blockers, and antiplatelet drugs. Conversely, the nutritionally sufficient group had significantly higher values of body mass index, hemoglobin, albumin, total cholesterol, LDL cholesterol, triglycerides, and pretreatment ejection fraction. There were no significant differences between the two groups regarding onset to balloon time, muscle enzyme levels such as CK and troponin, and the amount of contrast agent used. Variables from Table 1 were used as independent variables, and the non-AKI/AKI status (non-AKI = 0 and AKI = 1) was used as a dependent variable in a univariate regression analysis (Table 2). The results indicated that the onset to balloon time, white blood cell count, lactic acid, AST, LDH, baseline BUN, baseline creatinine, malnutrition (CONUT score ≧ 5), CK, and ejection fraction were significantly associated with AKI (*p* < 0.05). A multivariate logistic regression analysis was conducted using the four indicators with significant differences in the univariate analysis—lactic acid, baseline creatinine, malnutrition, and ejection fraction—along with age, which is considered a risk factor for AKI in AMI patients undergoing PCI [13], as independent variables. The non-AKI/AKI status (non-AKI = 0 and AKI = 1) was used as the dependent variable (Table 3). In our multivariate analysis, lactic acid, baseline creatinine, and malnutrition were significantly associated with AKI in patients with AMI. A multivariate logistic regression analysis revealed that malnutrition (CONUT score ≥ 5) was independently associated with AKI. To verify the strength of this connection, sensitivity analyses were performed by building additional models that incorporated important clinical variables, such as age, contrast dose, baseline creatinine, lactic acid, and ejection fraction. In every model, malnutrition remained significantly associated with AKI (Table 4).

## 4. Discussion

This single-center retrospective study investigated the association between malnutrition, AKI, and prognosis in AMI patients undergoing PCI. Our findings indicate that patients assessed with malnutrition using the CONUT score had approximately 2.5 times higher odds of developing AKI during acute-phase treatment compared with those without malnutrition. Furthermore, patients with malnutrition were associated with poorer short-term outcomes. Previous studies have reported the incidence of AKI in AMI cases ranging from 15.1% [14] to 23% [15]. Our findings show a similar incidence of renal impairment to that previously reported. Consistent with past reports, the prognosis of AMI patients who develop AKI was unfavorable [8]. Previous predictors of AKI development in AMI cases have included age [13], elevated baseline serum creatinine levels [16], decreased left ventricular systolic function [17], contrast volume [18], use of an intra-aortic balloon pump [19], and congestive heart failure [19]. To mitigate potential confounding and selection bias, propensity score matching is commonly utilized in observational studies. However, we chose not to use this method in the current study because it would significantly decrease the sample size and, therefore, lower the statistical power. Instead, to address these known risk factors and enhance the robustness of our findings, we conducted sensitivity analyses that integrated clinically relevant predictors of AKI in patients with AMI undergoing PCI. These predictors included age, serum lactate (as an indirect measure of shock), left ventricular ejection fraction, baseline serum creatinine, contrast medium volume, and nutritional status. In all models, malnutrition consistently emerged as an independent predictor of AKI. Based on these findings, our study identified malnutrition assessed by the CONUT score as a novel predictor of AKI development in AMI cases undergoing PCI, in addition to previously recognized risk factors.

While our study focused on the nutritional status and AKI in AMI cases undergoing PCI, previous reports, not limited to AMI cases, have documented relationships between malnutrition assessed by the mNUTRIC score and AKI development in PCI cases [20], as well as associations between malnutrition assessed by the PNI score and AKI [21]. In addition, the GNRI has been linked to the risk of AKI in ICU patients with acute heart failure [22]. While GNRI serves as a valuable nutritional risk index, its reliance on precise body weight measurements can restrict its utility in emergencies where immediate PCI is prioritized, particularly in AMI patients. Likewise, scoring systems like the mNUTRIC, which depend on various factors and require knowledge of comorbidities, might not be appropriate for swift clinical evaluations. Conversely, nutritional indices obtained solely from standard blood tests may be more advantageous for AMI patients undergoing urgent PCI. Multiple studies suggest that the PNI is a reliable measure of AKI risk in STEMI patients undergoing primary PCI [23, 24]. In addition, the CONUT score used in our research could serve as a practical and effective method for assessing AKI risk in this population, similar to the PNI. Furthermore, when compared with other nutritional or prognostic indices, such as the Naples Prognostic Score [25] or newer machine learning models [26], the CONUT score has the advantage of simplicity, as it depends solely on routine blood parameters. If it can consistently predict AKI, it would be particularly suitable for use in emergency settings.

While this study revealed a potential association between malnutrition and AKI development, the mechanisms underlying the relationship between the CONUT score and AKI onset were not fully elucidated. However, albumin, which is weighted twice compared to other CONUT components, has previously been implicated in AKI onset. Albumin is known to improve renal autoregulation by stabilizing endothelial cells, correlating with AKI [27]. Basic research has shown that albumin inhibits apoptosis of renal tubular cells through lysophosphatidic acid carriage and scavenging reactive oxygen species [28]. Clinical evidence supporting these mechanisms was highlighted in a 2017 meta-analysis [29], suggesting that low serum albumin levels may be associated with AKI onset in cardiac surgery or acute coronary intervention cases. A meta-analysis investigating serum albumin levels and AKI onset reported that with each 10 g/L decrement in serum albumin, the odds of AKI increased by 134% [30], indicating that low albumin levels directly influence AKI onset. Lymphocytes, another CONUT component, are also associated with AKI. Basic research has shown their roles in tubular repair and protection [31], and their anti-inflammatory effects [32]. Clinically, low lymphocyte counts have been linked to AKI onset and in-hospital mortality in cardiac surgery cases [33]. The final CONUT component, total cholesterol, is a malnutrition parameter included in various nutritional screening tools [34]. Although the precise mechanisms linking hypocholesterolemia and kidney injury are not well understood, omega-3 fatty acid-derived resolvins and protectins have been reported to block renal inflammation and suppress renal interstitial fibrosis [35]. In conclusion, low levels of albumin, lymphocytes, and cholesterol, which comprise the CONUT score, may each be associated with kidney injury. Beyond the possibility that each factor individually contributes to kidney injury, it is also possible that malnutrition itself is linked to the upregulation of inflammation in patients with renal dysfunction [36]. Furthermore, studies using various nutritional indicators have reported that malnutrition is a risk factor for AKI [37], suggesting that malnutrition itself may be directly associated with the onset of AKI.

Considering a report that 50%–60% of acute coronary syndrome cases were in a state of malnutrition [38], and in light of our current findings, patients with coronary artery disease could be at high risk of AKI due to their nutritional status. Given the limited time before initiating treatment for AMI patients undergoing PCI, a simple and rapid nutritional assessment tool is useful. There may be other nutritional indicators that are simpler, quicker, and more sensitive than the CONUT score for assessing the risk of AKI. Therefore, it is necessary to explore the most suitable indicator for evaluating the nutritional status of AMI patients undergoing PCI in future studies. Clinical reports also suggest that administering 20% exogenous albumin to patients with preoperative serum albumin levels below 4.0 g/dL immediately before off-pump coronary artery bypass surgery increases urine output and suppresses the onset of AKI [39]. In sepsis-induced AKI rat models, glutamine administration has been shown to control oxidative stress and prevent AKI development [40]. Future research should verify whether accurately assessing the nutritional status and considering therapeutic interventions can contribute to the prevention of AKI onset.

## 5. Limitations

This retrospective observational study has several limitations. Initially, using the creatinine level at admission as a baseline might have resulted in an underestimated incidence of AKI, particularly in patients with existing renal impairment on arrival. Nonetheless, with a median onset-to-balloon time of 250 min, it is important to recognize the established delay between the onset of renal injury and the observable changes in serum creatinine levels [41]. The likely impact of this limitation on the overall findings is modest. In addition, this study did not consider the treatment itself, including variations such as hydration protocols, nephrotoxic drug usage, and other AMI therapies, which obscures the influence of these factors on AKI development in AMI patients undergoing PCI.

Furthermore, a notable difference in baseline medication use was noted between nutritionally sufficient and malnourished groups. These discrepancies may stem from underlying health conditions or age-related factors; however, their exact causes are unclear, as comorbidities were not thoroughly evaluated in this study. Finally, we enrolled consecutive patients to minimize selection bias; however, due to the retrospective, single-center design, we were unable to eliminate selection bias. To validate our findings, larger multicenter prospective studies are necessary.

## 6. Conclusions

Evaluating malnutrition using the CONUT score suggests its association with AKI development in AMI cases undergoing PCI. Given the higher mortality rate among AMI patients with malnutrition compared with those without, careful treatment is essential. Adequate management is also required to mitigate the risk of AKI during intensive care.

## Figures and Tables

**Table 1:** Baseline characteristics of 211 AMI patients.

Variables	Total (*N* = 211)	Nutritionally sufficient group (*N* = 180)	Malnutrition group (*N* = 31)	*p* value
Age (years)	68 (59–79)	67 (58–77)	77 (66–83)	0.003
Male gender	156 (74%)	136 (76%)	20 (65%)	0.196
Body mass index	23.5 (21.3–25.7)	23.7 (21.5–25.8)	22.4 (19.2–23.5)	< 0.001
Smoking	121 (57%)	109 (61%)	12 (39%)	0.023
Door-to-balloon time (min)	74 (59–94)	74 (59–94)	73 (59–95)	0.821
Onset-to-balloon time (min)	250 (164–414)	254 (165–427)	231 (160–390)	0.262
Contrast medium usage (mL)	120 (90–150)	125 (90–154)	100 (80–140)	0.040
Mortality	6 (2.8%)	2 (1.1%)	4 (12.9%)	< 0.001
Hemoglobin (g/dL)	13.5 (12.2–14.8)	13.8 (12.6–15.1)	11.2 (9.4–12.3)	< 0.001
White blood cell (/mm^3^)	9900 (7900–12800)	10050 (8000–13200)	8700 (6500–11400)	0.105
Platelet count (/mm^3^)	22.0 (17.8–25.9)	22.0 (18.8–25.9)	22.2 (13.5–26.5)	0.988
Lactic acid (mmol/L)	1.7 (1.2–3.0)	1.7 (1.2–2.9)	2.4 (1.0–4.4)	0.143
AST (IU/L)	40 (26–73)	40 (26–70)	35 (26–119)	0.221
LDH (IU/L)	243 (200–353)	240 (203–346)	270 (181–611)	0.093
Total bilirubin (mg/dL)	0.7 (0.5–0.8)	0.7 (0.5–0.8)	0.6 (0.5–0.8)	0.501
Albumin (g/dL)	3.8 (3.5–4.0)	3.9 (3.6–4.1)	3.0 (2.7–3.3)	< 0.001
BUN (mg/dL)	16.0 (13.1–20.0)	16.0 (13.0–19.4)	18 (15–29)	0.023
Creatinine (mg/dL)	0.75 (0.62–0.97)	0.75 (0.61–0.94)	0.90 (0.71–1.18)	0.035
AKI	50 (24%)	34 (19%)	16 (52%)	< 0.001
Total cholesterol (mg/dL)	182 (156–212)	188 (164–215)	141 (122–167)	< 0.001
HDL cholesterol (mg/dL)	47 (40–54)	47 (40–54)	44 (35–55)	0.200
LDL cholesterol (mg/dL)	118 (95–138)	122 (102–143)	87 (69–111)	< 0.001
Triglyceride (mg/dL)	98 (64–167)	111 (67–180)	69 (44–90)	< 0.001
Hemoglobin A1c (%)	6.0 (5.7–6.7)	6.0 (5.7–6.7)	5.9 (5.7–6.9)	0.964
CK (U/L)	196 (122–446)	211 (122–472)	153 (97–421)	0.166
CK MB (U/L)	18 (7–53)	19 (8–54)	16 (7–44)	0.346
Troponin (ng/mL)	0.094 (0.024–0.586)	0.073 (0.024–0.565)	0.251 (0.036–1.303)	0.253
Ejection fraction (%)	50 (40–59)	51 (42–60)	45 (34–53)	0.002
Previous medications				
ACE/ARB	71 (34%)	56 (31%)	15 (48%)	0.060
Calcium channel blocker	53 (25%)	40 (22%)	13 (42%)	0.019
β blocker	20 (9%)	14 (8%)	6 (19%)	0.042
Oral antidiabetic	34 (16%)	28 (16%)	6 (19%)	0.595
Statin	48 (23%)	41 (23%)	7 (23%)	0.981
Antiplatelet drugs	44 (21%)	31 (17%)	13 (42%)	0.002

*Note:* Data are reported as number (%) or median (IQR). ACE, angiotensin-converting enzyme inhibitor; AST, aspartate aminotransferase.

Abbreviations: AKI, acute kidney injury; ARB, angiotensin II receptor blocker; BUN, blood urea nitrogen; CK, creatine kinase; HDL, high-density lipoprotein; LDH, lactate dehydrogenase; LDL, low-density lipoprotein.

**Table 2:** Univariate analysis of factors associated with AKI in AMI patient.

Variables	OR	OR (95% CI)	*p* value
Age (years)	0.99	(0.97, 1.02)	0.842
Male gender	2.16	(0.94, 4.96)	0.068
Body mass index	1.03	(0.93, 1.13)	0.620
Smoking	1.04	(0.55, 1.97)	0.915
Door-to-balloon time (min)	1.01	(1.00, 1.02)	0.223
Onset-to-balloon time (min)	1.00	(0.99, 1.00)	0.034
Contrast medium usage (mL)	0.99	(0.99, 1.00)	0.258
Hemoglobin (g/dL)	0.92	(0.79, 1.06)	0.220
White blood cell (/mm^3^)	1.00	(1.00, 1.00)	< 0.001
Platelet count (/mm^3^)	0.98	(0.94, 1.00)	0.121
Lactic acid (mmol/L)	1.53	(1.30, 1.81)	< 0.001
AST (IU/L)	1.00	(1.00, 1.00)	0.005
LDH (IU/L)	1.00	(1.00, 1.00)	< 0.001
Total bilirubin (mg/dL)	0.91	(0.36, 2.29)	0.843
BUN (mg/dL)	1.07	(1.03, 1.12)	< 0.001
Creatinine (mg/dL)	5.48	(1.71, 17.50)	< 0.001
Malnutrition	4.58	(2.064, 10.16)	< 0.001
Triglyceride (mg/dL)	1.00	(1.00, 1.00)	0.953
Hemoglobin A1c (%)	0.99	(0.69, 1.41)	0.987
CK (U/L)	1.00	(1.00, 1.00)	0.010
CK MB (U/L)	1.00	(1.00, 1.00)	0.769
Troponin (ng/mL)	1.13	(0.97, 1.33)	0.123
Ejection fraction (%)	0.95	(0.93, 0.98)	< 0.001

*Note:* AST, aspartate aminotransferase.

Abbreviations: AKI, acute kidney injury; AMI, acute myocardial infarction; BUN, blood urea nitrogen; CI, confidence interval; CK, creatine kinase; LDH, lactate dehydrogenase; OR, odds ratio.

**Table 3:** Multivariate analysis of factors associated with AKI in patients with AMI.

Factors	OR	OR (95% CI)	*p* value
Age (years)	1.00	(0.96, 1.03)	0.900
Lactic acid (mmol/L)	1.57	(1.31, 1.88)	< 0.001
Creatinine (mg/dL)	7.40	(1.85, 29.59)	0.005
Malnutrition	3.72	(1.28, 10.8)	0.016
Ejection fraction (%)	0.98	(0.95, 1.01)	0.187

Abbreviations: CI, confidence interval; OR, odds ratio.

**Table 4:** Sensitivity analysis: multivariate logistic regression models evaluating the association between malnutrition and AKI.

Model	Variables included	OR for malnutrition (95% CI)	*p* value
Model 1	Lactate, baseline creatinine, EF, contrast volume, malnutrition	3.52 (1.23–10.70)	0.019
Model 2	Age, baseline creatinine, EF, contrast volume, malnutrition	4.10 (1.61–10.41)	0.003
Model 3	Baseline creatinine, EF, contrast volume, lactate, malnutrition	3.82 (1.41–10.35)	0.008
Model 4	EF, contrast volume, lactate, age, malnutrition	3.91 (1.34–11.39)	0.012
Model 5	Contrast volume, lactate, age, baseline creatinine, malnutrition	3.72 (1.27–10.80)	0.016

Abbreviations: CI, confidence interval; EF, ejection fraction; OR, odds ratio.

## Data Availability

Raw data were generated at Kurume University Hospital. The data used to support the findings of this study are available from the corresponding author upon reasonable request.
